# Phage display for targeting PCSK9

**DOI:** 10.1016/j.ebiom.2021.103267

**Published:** 2021-03-06

**Authors:** Nicola Ferri

Since the development of the theoretical use of antibodies as therapeutic agents by Paul Ehrlich [1] and following the key scientific breakthrough for the development of the hybridoma technology [2], monoclonal antibodies (mAbs) have had a profound impact on therapy. DNA recombinant technologies have been utilised to replace the murine sequences with their human counterpart leading to the development of first chimeric, then humanised, and finally fully human therapeutic antibodies. The use of mAbs has expanded exponentially during the last two decades and currently covers several therapeutic areas, such as oncology, autoimmune, cardiovascular, and inflammatory diseases.

In this article of *EBioMedicine*, Shuhua Tan and colleagues describe the development of a new mAb anti PCSK9 (proprotein convertase subtilisin/kexin type 9) by using a phage display human single-chain fragment variable (scFv) library for antibodies selection [3]. This new technology allows the identification of antibodies outside the immune system, avoiding the use of experimental animals.

The authors first selected an scFv with high affinity to PCSK9, then performed two rounds of *in vitro* affinity maturation processes to further improve its affinity to the target. Finally, they constructed the full-length Fc-silenced anti-PCSK9 antibodies by fusing two anti-PCSK9 scFv variants (AP2M18 and AP2M21) to a modified human IgG1 Fc fragment.

The interest in targeting PCSK9 is derived from the fact that before 2015, low-density lipoprotein cholesterol (LDL-C) concentration could be lowered by reducing cholesterol intake in the diet (< 300 mg/day), and by using oral pharmacological therapies, *i.e.* statins, ezetimibe, and bempedoic acid. Statins treatment is associated with a 10–12% lower cardiovascular (CV) events per mmol/L reduction in LDL-C during the first year of treatment, followed by a 22–24% during each subsequent year [4]. Combination therapy with the Niemann-Pick C1-Like 1 (NPC1L1) inhibitor, ezetimibe, or the ATP-citrate lyase inhibitor, bempedoic acid, further control LDL-C levels and atherosclerotic cardiovascular disease (ASCVD) events in high-risk populations [5].

As highlighted by the EUROASPIRE, results from the most recent DA VINCI study [6] clearly documented that among patients with ASCVD, only 30% reached LDL-C levels <1.8 mmol/L and 18% - less than one in five - achieved LDL-C levels <1.4 mmol/L, as recommended by the 2019 ESC/EAS guidelines.

In 2003, Abifadel *et al* identified mutations in the gene *PCSK9* that cause autosomal dominant hypercholesterolemia. This finding has been leveraged into the most important therapy for the treatment of hypercholesterolemia and ASCVD since the introduction of the statins more than 30 years ago.

PCSK9 binds directly to the EGF-A domain of LDL receptor leading to its internalisation and lysosomal degradation. Thus, to block the protein-protein interaction between PCSK9 and the LDL receptor, the use of mAbs seemed to be the best approach to be used. This changed the paradigm of cholesterol treatment from oral to injectable and from daily to once or twice monthly dosing.

In 2015, two subcutaneously injectable mAbs, evolocumab and alirocumab, were approved for clinical use. Results from phase III clinical trials documented a potent hypocholesterolemic effect (-60% *vs* placebo) associated to a 15% lower CV risk.

Thus, PCSK9 represents an important pharmacological target for controlling hypercholesterolemia and its inhibition with mAbs seems the most effective approach for reducing CV risk. The antibody FAP2M21, described in this article of *EBioMedicine* binds to human PCSK9 with a KD as low as 1.42 nM, and showed a very slow dissociation rate (k_off_, 4.68 × 10^−6^ s^-1^). In a murine model of hypercholesterolemia expressing human PCSK9, FAP2M21 showed similar efficacy and potency than alirocumab, a result that was not trivial since the former binds the module 2 (amino acids 530-605) of the C-terminal domain (CTD) of PCSK9, while alirocumab blocks the interaction between the catalytic domain of PCSK9 and the EGF-A of the LDL receptor

[7]. Similarly, evolocumab interacts directly with residues from both the prodomain and catalytic domain of PCSK9 [8]. Thus, the development of FAP2M21 that inhibits PCSK9 with a different site of action than alirocumab and evolocumab may be useful for the treatment of patients resistant to these therapies. Although it is very rare to have non-responders to evolocumab and alirocumab (LDL-C reduction < 10% from baseline), some degree of resistance, with less than 30% reduction, have been observed [9].

By using this new technology, Shuhua Tan and colleagues have also been able to select an appropriate Fc fragment without immune effector functions. For therapies, anti PCSK9 Fc effector function should be avoided and IgG2 or IgG4 Fc fragments would be the optimal choice. However, both isotypes tend to aggregate and show lower conformational stability compared to IgG1, and IgG2 Fc is not completely devoid of antibody-dependent cellular cytotoxicity (ADCC). The authors chose a modified IgG1 Fc fragment without effector functions, such as ADCC and complement-dependent cytotoxicity activities. This molecular characteristic represents another level of differentiation between FAP2M21 and alirocumab, or evolocumab, which are, respectively, IgG1 and IgG2 antibodies. How these aspects will have a clinical impact on the pharmacokinetic and pharmacodynamic profile of FAP2M21 is unknown, but certainly represent an important aspect to investigate.

Taken together, by using the phage display-library technology the authors have developed a new anti PCSK9 mAb that can potentially be different than the previous two. Its possible differentiation is an important aspect to consider, not just from a marketing perspective, but also for offering a new therapeutic option for ASCVD patients in addition to the siRNA-GalNAc (N-acetylgalactosamine carbohydrates) anti-PCSK9 inclisiran, recently approved for clinical use [10].

## Declaration of Competing Interest

The author declares no conflict of interest.
